# Effects of wheat flour particle size on flour physicochemical properties and steamed bread quality

**DOI:** 10.1002/fsn3.2008

**Published:** 2021-07-16

**Authors:** Jinyue Pang, Erqi Guan, Yuling Yang, Mengmeng Li, Ke Bian

**Affiliations:** ^1^ College of Food Science and Engineering Henan University of Technology Zhengzhou China; ^2^ Henan Food Crop Collaborative Innovation Center Zhengzhou China

**Keywords:** flour quality, milling intensity, particle size, steamed bread

## Abstract

In this study, differently sized particles of wheat flour (from 52.36 μm to 108.89 μm) were obtained by adjusting the distance between the rolls (0.02, 0.04, 0.06, 0.08, and 0.1 mm) of a heart mill. Results showed that reducing the particle size significantly increased the damaged starch (DS) content. Uniaxial tensile measurement of dough showed that reducing the particle size of wheat flour can effectively increase the maximum tensile resistance, but the extensibility reaches the maximum in samples at medium particle diameter (78 and 66 μm). Additionally, the ratio of dynamic moduli (*G*″/*G*′) decreased with a reducing particle size. The results of disulfide bond content, gluten microstructure, showed that finer flour granulation can strengthen the gluten network. The steamed bread (SB) making test showed that SB made from wheat flour of a smaller particle size had a significantly smaller specific volume than that made from a larger particle size. The texture profile analysis showed that with a decrease of wheat flour particle size, the hardness, chewiness of SB increased, the resilience decreased, and there was no significant difference in adhesiveness. Overall, the quality of SB made flour of medium particles (78 μm) is better.

## Graphical abstract

This study is to obtain wheat flour of different particle size by adjusting the distance between the milling rolls and to study the effect of different particle sizes of wheat flour on the rheological properties of dough and the quality of steamed bread.

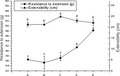

## 1 INTRODUCTION

Particle size has a significant effect on the functional properties of wheat flour. Previously, many studies have shown that particle size can significantly affect the physicochemical properties, such as water absorption, solvent retention, sedimentation, damaged starch (DS) content, falling values, and pasting properties, for example, and thus affect the rheological properties of the dough (water absorption, development time, etc) and the quality of the final products (specific volume of SB and bread, etc) (Kadan et al., 2010; León et al., 2006; Morrison et al., 1994; Rao et al., 2016). In the wheat milling process, the main factors affecting the particle size of wheat flour are milling equipment and methods including mechanical force and milling intensity. The mechanical force exerted by different equipment on wheat flour is different, so the particle size of wheat flour will be different. The same equipment, adjust different parameters or different processing time, will also affect the particle size of wheat flour. Many studies have been conducted on the effects of different milling equipment on the particle size and functional properties of wheat flour or whole wheat flour. For example, jet milling can significantly reduce the particle size of wheat flour; an increase in the jet‐milled fraction in wheat flour gave breads a lower specific volume, a lower crust lightness, and a higher bread hardness (Vouris et al., 2018). Protonotariou et al. (2014) found that jet milling had a noticeable effect on the characteristics of wheat flour; it had significantly higher water holding capacity, and the color of the wheat flour was improved. The temperatures and DS content generated during milling of wheat in stone, plate, hammer, and roller mills were different. Moreover, a greater loss of total amino acids was observed in the stone and plate milled flours when compared with that of hammer and roller milled samples (Prabhasankar & Rao, 2001). Some studies into the effects of different milling methods on the physical and chemical properties of wheat flour or whole wheat flour have been conducted. Okada et al. (1986) found that milling the same wheat flour an increased number of times can significantly increase the content of DS. Ma et al. (2016) found that treating wheat flour with a ball mill for different times or adjusting the rotation speed resulted in wheat flour with different DS content (9.3%–30%), thus affect the farinographical properties of flour, and changed the pasting properties. Wang et al. (2016) changed bran particle size by milling it for different times and then added it back to the wheat flour to obtain reconstituted whole wheat flour of different particle sizes. It was found that the SB made from the smaller‐sized whole wheat flour has a larger specific volume, smaller cells, and better taste compared with the larger particle size (Wang et al., 2017).

SB is a traditional Chinese staple food and is widely consumed in many Asian countries. The studies referenced above show that particle size has a great influence on the physical and chemical properties of wheat flour and the quality of SB. However, there has been research on the influence of particle size difference on food quality due to milling intensity. Therefore, the purpose of this study is to study the effect of different particle sizes of wheat flour on the rheological properties of dough and the quality of SB, so as to provide theoretical basis for the adjustment of flour milling parameters for SB flour.

## 2 MATERIALS AND METHODS

### 2.1 Raw material and preparation of sample

Granular wheat flour was purchased from Tianfeng Flour Co., Ltd. in Henan province, and the granular flour was taken from the 2P undersieve material in the production line of the flour mill. Experimental Mill (MLU‐202, Wuxi Buhler Machinery Manufacturing Co., Ltd., China) was used to produce different milling intensity wheat flour.

In this experiment, samples of five different particle sizes were obtained by milling the 2P undersize material by adjusting the distance between the rolls (A: 0.1 mm, B: 0.08 mm, C: 0.06 mm, D: 0.04 mm, E: 0.02 mm) of a heart mill using a Buhler experimental mill. The grain does not pass through any mesh sieve during grinding.

Dry method was used for particle size determination. The particle size distribution and as equivalent diameters at cumulative volumes of 10% (*D*_10_), 50% (*D*_50_), and 90% (*D*_90_) were determined using a laser particle size analyzer (Mastersizer 2000, Malvern). The DS content of five samples was determined according to the AACC International Approved Methods 76‐30A.

### 2.2 Dough rheology

The uniaxial tensile strength of dough was determined according to the method described by (Londono et al., 2013). First, 300 g wheat flour and 159 g distilled water were mixed in a dough mixer (Needle dough mixer) for 10 min to form dough. Then, the dough was placed in a constant temperature and humidity proofing box (temperature 30°C, humidity 90%) for 20 min. The dough was made into rectangular blocks with a matching mold of texture analyzer. The uniaxial tensile properties of the dough were measured using a TA‐XTplus Texture Analyzer A/KIE probe (Stable Microsystems). The pretest, test, and postmeasure speeds were 2.0, 3.3, and 10.0 mm/s, respectively, with a test distance of 50 mm and a triggering force of 5 g. Seven parallels samples were made for each condition.

### 2.3 Determination of dynamic rheological characteristics of dough

The dynamic rheological characteristics of dough were determined according to Yazar et al. (2016). The sample preparation was conducted as for the uniaxial tensile measurement. A small piece of dough was placed on a high‐grade rotary rheometer (MARS 60, HAAKE Co., Ltd) test platform with a diameter of 35 mm and a parallel plate spacing of 2 mm. The parallel plate was moved; then, the dough pressed to 2 mm thick, the excess cut off, and the outer edge of the dough sealed with mineral oil. After the dough was relaxed on the parallel plate for 5 min, the strain‐scanning procedure in the dynamic measurement mode determined the linear viscoelastic region of the dough. The dynamic rheological properties of wheat dough were measured using the frequency scanning program, in which the strain was 0.1%, the temperature was 30°C, and the frequency scanning range was 0.1–10 Hz. Each sample was tested 3 times in parallel.

### 2.4 Disulfide bond content and free sulfhydryl content

Disulfide bond content and free sulfhydryl content were prepared according to the method of Beveridge et al. (1974) with minor revisions. Method for determination of free sulfhydryl (SH) content: Weigh 0.01 g of lyophilized powder of dough, add 0.5 ml of distilled water to make a mixed solution, then add 2.5 ml of Tris‐Gly‐8M Urea, and then add 0.02 ml of DTNB solution (4 mg/ml). Let stand at room temperature for 30 min. Finally, use a spectrophotometer to measure the absorbance of the supernatant at 412 nm, at the same time determine the blank sample, and simultaneously determine 3 parallels. The calculation formula is as follows:

$$\mu \text{mol}\;\text{SH}/g=73.53\times A_{412}\times D/C$$

In the formula, *A*_412_ is the absorbance at *λ* = 412 nm; *D* is the dilution factor; and *C* is the sample concentration.

Method for determination of disulfide bond (S‐S) content: Weigh 0.01 g of lyophilized dough powder, add 0.5 ml of distilled water to make a mixed solution, add 0.05 ml of mercaptoethanol, 2 ml of urea, and after standing for 1 hr, add 5 ml of trichloroacetic acid (TCA) and continue to stand for 1 hr, then centrifuge at 5,000 r/min for 10 min, repeat washing twice with 5 ml of TCA, add 5 ml of urea (8 mol/L) and Ellman's reagent to the precipitate, take the supernatant and measure the absorbance of the sample at 412 nm. The blank group is 5 ml urea, and the samples of each group are measured 3 times, and the average value is taken as the final result. The calculation formula is as follows:

$$\mu \text{mol}\;\left(\text{total}\;\text{SH}\right)/g=73.53\times A_{412}\times D/C$$

$$\mu \text{mol}\;\left(S‐S\right)/g=\left(N_{2}‐N_{1}\right)/2$$

*N*_2_ represents the total sulfhydryl content in the protein after the disulfide bonds are reduced; *N*_1_ represents the free sulfhydryl content.

### 2.5 Dough microstructure

The dough preparation was conducted as for the uniaxial tensile measurement. Prepare the sample dough into a cube of about 2.5 cm, and then freeze it in a refrigerator at −30°C for 3 hr. The frozen dough was sliced with a microtome cryostat and stained with 0.001 g/L Rhodamine B solution (Lucas et al., 2018). The slides carrying the samples were covered with coverslips; then, the observation of microstructure of dough samples was applied using a confocal scanning laser microscope (FIUOVIEW FV300, China) in ×40 objective. For visualization, a laser with a wavelength of 561 nm was used. The ranges of fluorescence emission of Rhodamin B for protein were 545–660 nm.

### 2.6 SB preparation

SB was prepared according to the method of Sun et al. (2007) with minor revisions. Dry yeast (1.6 g) was weighed and dissolved in 50 ml of distilled water at 38°C for use. Next, 200 g of wheat flour was added to a blender, with the yeast liquid. An appropriate amount of distilled water was then added, 70%–80% of the water absorption rate of the powder. The dough was formed after stirring for 10 min in the blender and then immersed in a fermentation tank at 30°C and 85% RH for 30 min. It was then steamed for 25 min, cover the dry gauze after reaching room temperature and cool for 1 hr.

### 2.7 Quality evaluation of SB

The color of the SB epidermis was quantified by using a colorimeter (CE‐410, Konica Minolta), which was calibrated by using white and black standard bricks before use.

The specific volume is expressed in ml/g. The cooled SB was weighed, and the volume was determined by the millet replacement method (Cao et al., 2019). The textural profile analysis of SB was determined using the Texture Analyser (Model: TA‐XT2i, Stable Microsystems) equipped with a 25 mm diameter aluminum cylindrical probe with pretest speed 3 mm/s, test speed 1 mm/s, and post‐test speed 5 mm/s. The deformation level was 50% of the original height. Hardness, springiness, cohesiveness, and chewiness were calculated.

### 2.8 Sensory evaluation

Sensory evaluation was conducted according to the methods of Wronkowska et al. (2015) with slight modification. The group consisted of 20 trained panelists (9 males and 11 females, aged between 22 and 27) from different provinces in China. The panelists assigned a score (1:low–9:high) for each quality attribute such as appearance, texture, taste, elasticity, and overall acceptance.

### 2.9 Statistical analysis

All tests were performed in at least triplicate. All the data obtained in this study were expressed as mean ± standard deviation (*SD*). *p* < .05 was used to define the significance of differences between the samples. One‐way analysis of variance and Duncan's multiple‐range tests were performed using SPSS 16.0 for windows (SPSS Inc.). All the diagrams were drawn with Origin 8.5.

## 3 RESULTS AND DISCUSSION

### 3.1 Particle size distribution and median particle diameter of wheat flour

The particle size distribution of five samples is shown in Table 1. The trends of the changes in the flour particle size parameters including *D*_10_, *D*_90_ are similar to that of *D*_50_. For different milling intensity, *D*_50_ values of the A, B, C, D, and E were 108.89, 88.13, 78.47, 65.73, and 52.36 μm, respectively. The lowest value of *D*_50_ was obtained for E, indicating that the smaller the distance between the milling rolls, the greater the mechanical force the wheat granules are subjected to, thereby reducing the particle size of the wheat flour.

**TABLE 1:** Particle size distribution and DS content of wheat flour with different particle sizes

Samples	*D*_10_ (μm)	*D*_50_ (μm)	*D*_90_ (μm)	DS content (%)
A	48.46 ± 0.02^a^	108.89 ± 0.21^a^	183.68 ± 0.17^a^	11.73 ± 0.15^e^
B	32.46 ± 0.70^b^	88.13 ± 0.57^b^	157.07 ± 0.78^b^	17.47 ± 0.15^d^
C	26.23 ± 0.49^c^	78.47 ± 1.27^c^	141.76 ± 1.67^c^	20.57 ± 0.15^c^
D	20.41 ± 0.47^d^	65.73 ± 0.59^d^	123.51 ± 0.90^d^	24.43 ± 0.38^b^
E	17.02 ± 0.41^e^	52.36 ± 1.34^e^	110.09 ± 1.83^e^	28.80 ± 0.20^a^

Note

Data are expressed as the mean ± standard deviation. Values in the same column with different letters are significantly different at *p* < .05.

Abbreviation: DS, damaged starch.

### 3.2 Effect of particle size on DS content and dough uniaxial extension of wheat flour

As shown in Table 1, reducing particle size significantly increased the DS content in wheat flour. During the milling of wheat, some starch granules sustain mechanical damage. The level of damage depends on the hardness of the wheat and milling technology used (Barrera, Bustos, et al., 2013). In this experiment, the increase in DS content was due to the mechanical force upon the wheat granules during the milling process increases. In previous work, it was found that the Rapid Visco Analyzer (RVA) pasting properties in wheat flour were affected by the amount of DS (León et al., 2006). When the content of DS increases, the peak viscosity, final viscosity, breakdown, and setback gradually decrease (Barrera, Bustos, et al., 2013). An appropriate amount of DS (12.2%–21.9%) can improve the quality of bread (Boyacı et al., 2004; Ma et al., 2016). However, too high levels of DS can cause a decline in the quality of the bread and SB (Barak et al., 2014; Liu et al., 2014).

**FIGURE 1:**
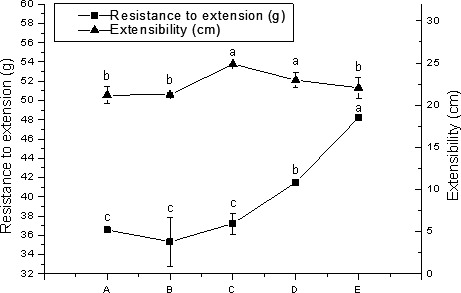
Resistance of wheat flour samples with different particle sizes to extension and extensibility. ^a^Data were expressed as the mean ± standard deviation. Values in the same column with different letters were significantly different at *p* < .05 (A: *D*_50_ = 108.89 μm; B: *D*_50_ = 88.13 μm; C: *D*_50_ = 78.47 μm; D: *D*_50_ = 65.73; E: *D*_50_ = 52.36 μm)

Uniaxial extension testing is commonly used to study the behavior of dough. In this study, the variation of uniaxial tensile properties of dough prepared with different particle sizes is shown in Figure 1. After the particle size of the wheat flour was reduced, we found that the maximum tensile resistance of the dough increased, but the extensibility reached a maximum in sample C and sample D. Dough strength and extensibility are reported to be positively correlated with SB volume (He et al., 2003). Suitable dough extensibility is beneficial for the production of SB, which is flexible enough to avoid rupture of the gas cells during the proofing of the SB (Zhang et al., 2014). In this experiment, sample E with the smallest particle size had the largest maximum tensile resistance, but the extensibility was reduced, which is disadvantageous for the specific volume of the SB.

### 3.3 Effect of particle size on dynamic rheological characteristics of dough

**FIGURE 2:**
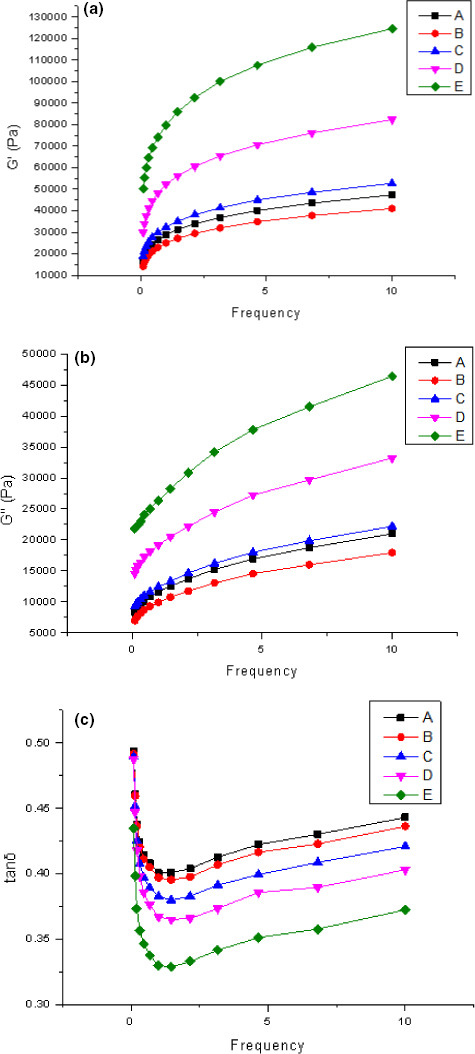
Frequency sweep of wheat flour samples of different particle size wheat flour. (a) Storage modulus (*G*′); (b) loss modulus (*G*″); (c) tan δ (*G*″/*G*′) (A: *D*_50_ = 108.89 μm; B: *D*_50_ = 88.13 μm; C: *D*_50_ = 78.47 μm; D: *D*_50_ = 65.73; E: *D*_50_ = 52.36 μm)

*G*′ is the storage modulus, which characterizes the energy stored in the material after elastic deformation, and is positively correlated with elasticity of the object; *G*″ is the loss modulus, which indicates the amount of energy lost by the viscous deformation of the material, and is positively correlated with the viscosity (Skendi et al., 2011). Tan δ is the ratio of *G*″ to *G*′, which can be used to characterize the relative size of viscous modulus and elastic modulus, that is, whether the material tends to be viscous or elastic. In this study, for all samples, *G*′ was larger than *G*″ over the range of test frequencies, indicating that the elastic characteristics of the dough were more prominent than the viscous characteristics. Dough from all the samples showed a decrease in the tan *δ* values with a decrease of the particle size of wheat flour (Figure 2c), which indicates the dough becomes more elastic. As the particle size decreased, both *G*′ (Figure 2a) and *G*″ (Figure 2b) increased, and both reached maximum when the wheat flour had the smallest particle size, except for sample A and sample B. This shows that as the particle size decreases, the elasticity of the dough increases. Part of the reason for the increase in dough elasticity (Figure 2) can be attributed to the increase in DS (Table 1) accompanying the wheat flour micronization process. DS has been found to spontaneously swell and gel, even when placed in cold water (Morrison et al., 1994), thus enhancing its elasticity and mechanical strength during dough formation. In addition, the gluten gel network strength during dough development can be enhanced by an increased particle surface area (Lazaridou et al., 2018). Similar conclusions appeared as early as 1999, Shim and Mulvaney (1999) reported that the balance between the intact starch granules and gelatinized or damaged ones affected *G*′ values.

### 3.4 Disulfide bond content

**FIGURE 3:**
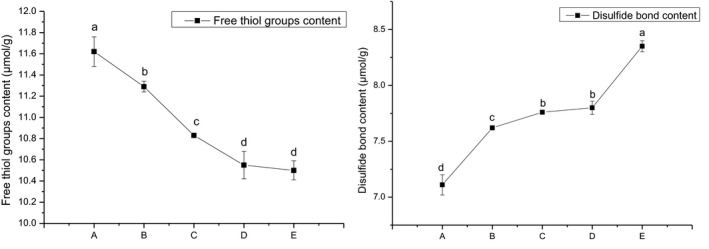
Free sulfhydryl groups and disulfide bond contents of wheat dough powder with different particle sizes. ^a^Data were expressed as the mean ± standard deviation. Values for the same parameter with different letters were significantly different at *p* < .05 (A: *D*_50_ = 108.89 μm; B: *D*_50_ = 88.13 μm; C: *D*_50_ = 78.47 μm; D: *D*_50_ = 65.73; E: *D*_50_ = 52.36 μm)

For wheat gluten, the most important chemical bond to maintain its molecular structure stability is the S–S content and its exchange reaction with SH (Lamacchia et al., 2011). Free SH, as functional groups on the surface of protein molecules, has important effects on protein cross‐linking and gel formation (Campbell et al., 2009). Free SH can form S–S through oxidation. This process directly affects the formation and strength of the protein network structure. Therefore, the content of free SH can directly indicate the content of S–S (Grosch & Wieser, 1999). In this study, as the particle size decreased, the free SH in wheat dough flour gradually decreased, and the S–S content gradually increased (Figure 3). This phenomenon indicates that after the wheat flour particles become finer, the specific surface area of the particles increases, and free SH in contact with oxygen in the air increases, so that the possibility of free SH is converted into S–S increased, and finally the content of free SH decreases and the content of S–S increases (Guo et al., 2017). In this experiment, the dough with the smallest particle size has the best viscoelasticity and maximum tensile resistance (Figures 1 and 2). On the one hand, it may be due to the proper increase of the DS content, which increases the mechanical strength of the dough (Angelidis et al., 2016); on the other hand, it may be the increase of the S–S content, which strengthens the gluten network structure. Studies have shown that an increase in S–S in proteins will promote protein cross‐linking and strengthen gluten structure (Lamacchia et al., 2011).

### 3.5 Gluten microstructure

**FIGURE 4:**
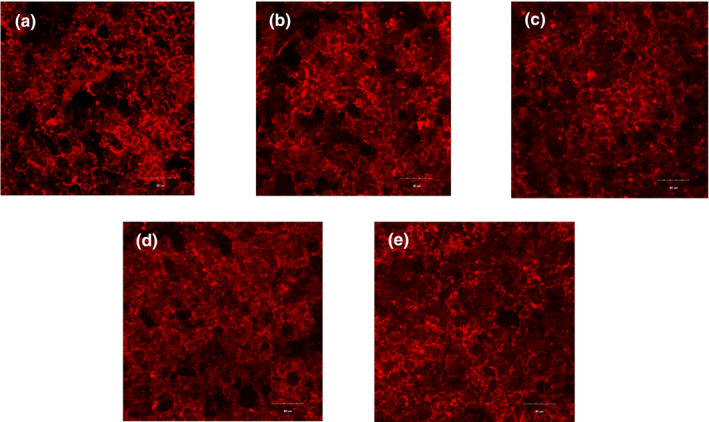
Microstructure of dough produced with different particle size wheat flour. (A: *D*_50_ = 108.89 μm; B: *D*_50_ = 88.13 μm; C: *D*_50_ = 78.47 μm; D: *D*_50_ = 65.73; E: *D*_50_ = 52.36 μm)

LSM is a new type of microscopic imaging technology. In recent years, it has been more and more applied to the food research field (Ferrando & Spiess, 2016). Among them, the microstructure of flour products is used more frequently (Dürrenberger et al., 2001). The red part in Figure 4 is the protein that binds to rhodamine B. It can be seen from Figure 4 that sample A has the most black voids, indicating that the gluten network structure is the worst and the protein network structure is not dense. As the particle size of wheat flour decreases, the gluten network structure becomes more and more continuous and uniform, and sample E is the densest, which is consistent with higher S–S content. The experimental results show that as the particle size of wheat flour decreases, the network structure of the dough becomes denser, which is mainly related to the increase of the S–S content, and is consistent with the results determined by the dynamic rheological characteristics and maximum tensile resistance characteristics.

### 3.6 Effect of particle size on color and specific volume of SB

The color of SB was significantly affected by different particle size as presented in Table 2. The L* values (lightness) varied from 92.39 to 93.53, with highest value for sample E and the lowest for sample A. As the particle size of wheat flour decreased, the L* of SB significantly increased. This may be due to the smaller particle having fine pores and good uniformity, which reduces the light absorption ability and enhances the light reflection ability, thus increasing the whiteness of the SB. The a* values (±, redness/greenness) ranged from −2.33 to −2.58, and the b* values (±, yellowness/blueness) ranged from 21.99 to 24.16, which indicated that the particle size can also affect the red, green values and the yellow, blue values of the surface of the SB.

**TABLE 2:** Color and specific volume of steamed bread of wheat flour from different particle size

Samples	L*	a*	b*	Specific volume (ml/g)
A	92.39 ± 0.02^b^	−2.38 ± 0.02^b^	21.99 ± 0.33^b^	1.90 ± 0.04^d^
B	92.38 ± 0.01^b^	−2.33 ± 0.04^b^	22.27 ± 0.10^b^	2.04 ± 0.02^bc^
C	92.20 ± 0.37^b^	−2.51 ± 0.08^a^	22.85 ± 0.53^b^	2.10 ± 0.02^a^
D	92.42 ± 0.34^b^	−2.56 ± 0.07^a^	22.55 ± 0.34^b^	2.00 ± 0.07^c^
E	93.53 ± 0.35^a^	−2.58 ± 0.07^a^	24.16 ± 0.97^a^	2.01 ± 0.03^c^

Note

Data were expressed as the mean ± standard deviation. Values in the same column with different letters were significantly different at *p* < .05 (A: *D*_50_ = 108.89 μm; B: *D*_50_ = 88.13 μm; C: *D*_50_ = 78.47 μm; D: *D*_50_ = 65.73; E: *D*_50_ = 52.36 μm).

**FIGURE 5:**
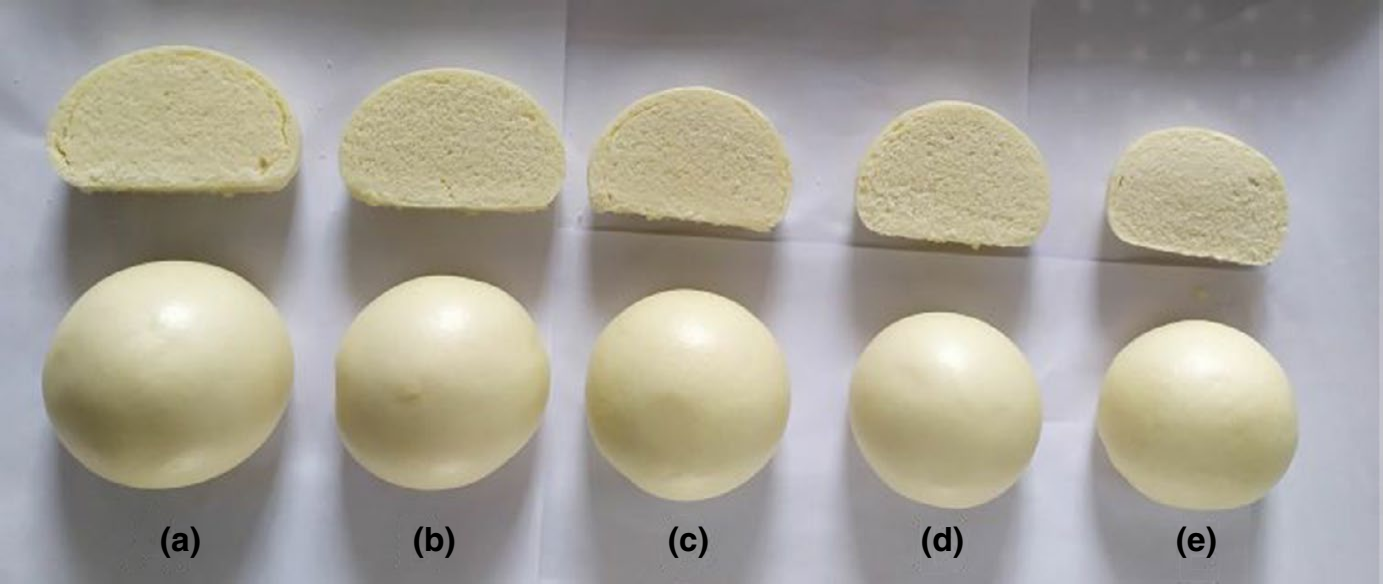
Images of SB made by different particle size wheat flour. (A: *D*_50_ = 108.89 μm; B: *D*_50_ = 88.13 μm; C: *D*_50_ = 78.47 μm; D: *D*_50_ = 65.73; E: *D*_50_ = 52.36 μm)

The specific volume is one of the most important indicators to measure the quality of SB, which can greatly influence the consumer's choice (Niu et al., 2017). The SB made from wheat flour of different particle sizes is shown in Table 2. First an increase and then a decrease in specific volume were observed with a decrease in particle size of wheat flour, which is the same as the trend of extensibility of tensile properties. Part of the reason may be that the gluten network is too close, which causes the volume of the steamed bread to become smaller (Figure 5), and another part may be due to the increase in the content of DS. DS content is an important factor affecting the specific volume of SB. Ma et al. (2016) found that an increase in DS content significantly reduced the specific volume of SB. In this experiment, due to the increase of the milling intensity, the starch crystallized area was destroyed; thus, the DS content increased (Barrera, Calderón‐Domínguez, et al., 2013). DS is easily broken down into maltose by amylase; maltose is the food material of yeast during dough fermentation. Proper enzymatic hydrolysis can improve hydration and increase the extensibility of the gas chamber wall of the dough, thus resulting in a much larger product during cooking and baking (Boyacı et al., 2004). However, an excessive enzymatic reaction produces a large amount of residual low sugar and dextrin, so that the remaining portion is insufficient to combine the moisture in the dough during the gelatinization process. An increase in the viscosity of the dough center causes the internal texture to be too soft to support a large volume during cooking or baking, thus the final specific volume of steamed bread is too small (Wang et al., 2001).

### 3.7 Effect of particle size on texture properties of SB

The effect of particle size on SB quality is shown in Table 3. For SB, a soft texture is ideal. As the particle size of wheat flour decreases, the hardness increases. It has been reported that crumb hardness and specific volume are negatively correlated (Ronda et al., 2015). This was also true in this experiment; smaller particle size resulted in a smaller specific volume, and the hardness of the prepared SB was also larger. Similar studies have drawn similar conclusions, and Protonotariou et al. (2015) reported that bread made from jet‐milled fractions of whole wheat had harder crumbs than the control sample.

**TABLE 3:** Textural profile analysis of SB of wheat flour from different particle size

Samples	Hardness (g)	Springiness	Chewiness (g)	Resilience	Adhesiveness
A	2,864.94 ± 242.85^b^	0.92 ± 0.00^bc^	2,226.62 ± 181.33^b^	0.47 ± 0.00^a^	15.18 ± 2.75^a^
B	2,954.27 ± 131.36^b^	0.89 ± 0.00^d^	2,226.46 ± 71.11^b^	0.47 ± 0.00^a^	17.00 ± 1.38^a^
C	3,136.51 ± 296.53^b^	0.91 ± 0.01^cd^	2,374.53 ± 234.79^b^	0.47 ± 0.00^a^	4.94 ± 3.40^a^
D	4,280.23 ± 103.48^a^	0.94 ± 0.01^a^	3,304.70 ± 59.71^a^	0.46 ± 0.00^b^	14.82 ± 12.10^a^
E	4,574.05 ± 201.45^a^	0.93 ± 0.00^ab^	3,500.64 ± 121.48^a^	0.44 ± 0.01^c^	3.60 ± 0.45^a^

Note

Data were expressed as the mean ± standard deviation. Values in the same column with different letters were significantly different at *p* < .05 (A: *D*_50_ = 108.89 μm; B: *D*_50_ = 88.13 μm; C: *D*_50_ = 78.47 μm; D: *D*_50_ = 65.73; E: *D*_50_ = 52.36 μm).

The chewiness value of SB increases with a decrease of wheat flour particle size. The resilience showed a downward trend, springiness was at a maximum value when wheat flour had a smaller particle size, but there was no significant difference in adhesiveness. Studies have shown that there is significant connection between the chewiness and hardness of SB (Ru et al., 2015). As shown in Table 3, the changes in the chewiness of SB exhibited the same trends as hardness. However, Wang et al. (2017) reported that the hardness, cohesiveness, and chewiness values of whole wheat flour SB decreased with a decrease in mill feed particle size. Protonotariou et al. (2016) reported that by adjusting the different conditions of the jet mill to obtain different particle sizes, intense milling increased the hardness of the batter and increased the interaction between the different components. It was observed that changing the particle size of wheat flour, changing the particle size of bran, or changing the particle size of whole wheat flour has different effects on the final product. In this experiment, the dough having with a smaller wheat flour particle size has a higher *G′* value and a *G*″ value, and higher proportion of elasticity to viscosity, but the inner structure is too tight, and the produced SB has a small volume and poor texture characteristics.

### 3.8 Effect of particle size on sensory evaluation of SB

**FIGURE 6:**
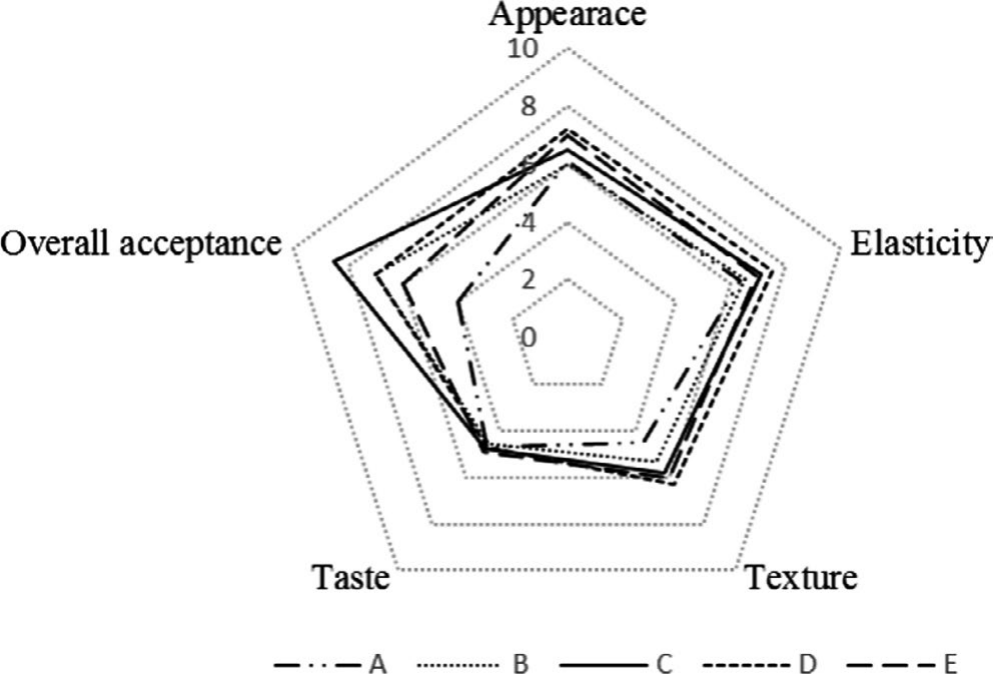
Sensory evaluation of wheat flour samples of different particle size wheat flour. (A: *D*_50_ = 108.89 μm; B: *D*_50_ = 88.13 μm; C: *D*_50_ = 78.47 μm; D: *D*_50_ = 65.73; E: *D*_50_ = 52.36 μm)

Results of sensory evaluation of SB are shown in Figure 6. As can be seen from the QDA plot, the overall acceptance of sample C and sample D was higher than other samples, while SB made from sample A received the lowest score. That is to say, if the particle size is too large or too small, the popularity of the SB will be reduced. In general, the particle size had a little effect on the taste of SB, but it had a greater impact on several other indicators. The elasticity of SB made by sample D was better than other samples. The texture of the SB made from sample A had the lowest score and sample D had the highest score. Figure 5 shows the appearance of SB. The results showed that that SB made from sample D had better appearance and internal structure than other samples, especially those made from sample A and sample B.

## 4 CONCLUSION

Particle size has a great influence on the physical and chemical properties of wheat flour and its final product. This work evaluated the influence of five milling intensities on the physicochemical properties and quality of wheat flour for SB. We found that an increase in the milling gap significantly reduced the particle size of the wheat flour and increased the DS content. Reduction of wheat flour average particle size from 108.89 to 52.36 μm effectively increased the maximum tensile resistance, but the extensibility reached a maximum in sample C (78.47 μm) and sample D (65.73 μm). Dough dynamic oscillation characteristics showed that the viscoelasticity of the dough improved remarkably as the particle size increased. The results of disulfide bond content, gluten microstructure, showed that increased milling strength can strengthen the gluten network. The SB making test showed that SB made from wheat flour of smaller particle size exhibited a lower specific volume, harder taste, and brighter SB surface. These results suggest that reducing the particle size of wheat flour from 108 to 52.36 μm can make the internal structure of the SB firm, resulting in a lower sensory score. Overall, the quality of SB made from sample C (78.47 μm) is better.

## CONFLICT OF INTEREST

None.

## AUTHOR CONTRIBUTIONS

**Jinyue Pang:** Conceptualization‐Equal, Data curation‐Equal, Formal analysis‐Equal, Funding acquisition‐Equal, Investigation‐Equal, Methodology‐Equal, Project administration‐Equal, Resources‐Equal, Software‐Lead, Supervision‐Equal, Validation‐Equal, Visualization‐Equal, Writing‐original draft‐Lead, Writing‐review & editing‐Lead. **Erqi Guan:** Conceptualization‐Equal, Data curation‐Equal, Formal analysis‐Equal, Funding acquisition‐Equal, Investigation‐Equal, Methodology‐Equal, Project administration‐Supporting, Resources‐Equal, Software‐Supporting, Supervision‐Equal, Validation‐Equal, Visualization‐Equal, Writing‐original draft‐Equal, Writing‐review & editing‐Equal. **Yuling Yang:** Data curation‐Equal, Methodology‐Equal, Software‐Equal. **Mengmeng Li:** Data curation‐Equal, Investigation‐Equal, Software‐Equal. **Ke Bian:** Conceptualization‐Equal, Data curation‐Equal, Formal analysis‐Equal, Funding acquisition‐Supporting, Investigation‐Equal, Methodology‐Equal, Project administration‐Supporting, Resources‐Supporting, Software‐Equal, Supervision‐Equal, Validation‐Equal, Visualization‐Equal, Writing‐original draft‐Equal, Writing‐review & editing‐Equal.

## COMPLIANCE WITH ETHICS REQUIREMENTS

This article does not contain any studies with human or animal subjects.

## Data Availability

All data, models, and code generated or used during the study appear in the submitted article.
